# Geometric complement heterogeneous information and random forest for predicting lncRNA-disease associations

**DOI:** 10.3389/fgene.2022.995532

**Published:** 2022-08-24

**Authors:** Dengju Yao, Tao Zhang, Xiaojuan Zhan, Shuli Zhang, Xiaorong Zhan, Chao Zhang

**Affiliations:** ^1^ School of Computer Science and Technology, Harbin University of Science and Technology, Harbin, China; ^2^ College of Computer Science and Technology, Heilongjiang Institute of Technology, Harbin, China; ^3^ Department of Endocrinology and Metabolism, Hospital of South University of Science and Technology, Shenzhen, China; ^4^ Hunan Provincial Key Lab on Bioinformatics, School of Computer Science and Engineering, Central South University, Changsha, China

**Keywords:** lncRNA-disease association prediction, geometric complement heterogeneous information, random forest, autoencoder, machine learning

## Abstract

More and more evidences have showed that the unnatural expression of long non-coding RNA (lncRNA) is relevant to varieties of human diseases. Therefore, accurate identification of disease-related lncRNAs can help to understand lncRNA expression at the molecular level and to explore more effective treatments for diseases. Plenty of lncRNA-disease association prediction models have been raised but it is still a challenge to recognize unknown lncRNA-disease associations. In this work, we have proposed a computational model for predicting lncRNA-disease associations based on geometric complement heterogeneous information and random forest. Firstly, geometric complement heterogeneous information was used to integrate lncRNA-miRNA interactions and miRNA-disease associations verified by experiments. Secondly, lncRNA and disease features consisted of their respective similarity coefficients were fused into input feature space. Thirdly, an autoencoder was adopted to project raw high-dimensional features into low-dimension space to learn representation for lncRNAs and diseases. Finally, the low-dimensional lncRNA and disease features were fused into input feature space to train a random forest classifier for lncRNA-disease association prediction. Under five-fold cross-validation, the AUC (area under the receiver operating characteristic curve) is 0.9897 and the AUPR (area under the precision-recall curve) is 0.7040, indicating that the performance of our model is better than several state-of-the-art lncRNA-disease association prediction models. In addition, case studies on colon and stomach cancer indicate that our model has a good ability to predict disease-related lncRNAs.

## 1 Introduction

Long non-coding RNA (lncRNA) is a kind of non-coding RNA with a length of more than 200 nucleotides, which have received increasing attention from researchers. LncRNAs have now been proved to play a key role in transcriptional and posttranslational regulation (Taft et al., 2010; Mathieu et al., 2014; Sun et al., 2018; Xie et al., 2018). The pathogenesis of a series of diseases is significantly associated with mutations and dysregulation of lncRNAs (Washietl et al., 2014; Chen et al., 2017). For example, MALAT1 was discovered to be overexpressed in many entity tumors such as lung cancer (Cheetham et al., 2013). It was shown that clonogenic and anchorage-dependent growth of lung cancer cells would be significantly decreased when H19 was down-regulated (Barsyte-Lovejoy et al., 2006). Confirming the associations between lncRNAs and diseases by biological experiments is time-consuming, labor-intensive and challenging, so using computational method to predict the associations not only provides a more efficient way for biological experiments but also reduces a lot of unnecessary human and material resources. Currently, dozens of computational models have been proposed to identify disease-associated lncRNAs based on various biological data. We can broadly classify the current computational models for lncRNA-disease association (LDA) prediction into three categories.

The first class of LDA prediction models is based on biological networks. Sun et al. implemented random walk and restart on lncRNA functional similarity network (Sun et al., 2014). Zhou et al. integrated the LDA network, disease similarity network and lncRNA-miRNA interaction network into a heterogeneous network and applied random walk on the network (Zhou et al., 2015). Chen et al. integrated the known LDAs, lncRNA expression profiles, lncRNA functional similarity, disease semantic similarity and Gaussian interaction profile kernel similarity to predict potential LDAs (Chen, 2015a). Ping et al. (2019) constructed a model based on the known LDA network. However, these models need the known LDA network. Thus, Liu et al. (2014) conceived a model by integrating the known human expression profiles of lncRNA and disease genes, which is the first computational model without relying on the known LDAs. Chen et al. combined miRNA-disease association and lncRNA-miRNA interactions to form a model called HGLDA (Chen, 2015c). Zhou et al. developed a computational method by integrating association among lncRNA, protein, disease, miRNA, drug and high-order proximity preserved embedding for predicting LDAs (Zhou et al., 2021). Sumathipala et al. used the topology of a multi-level network consisting of lncRNA-protein, protein-protein interactions and protein-disease associations to identify LDAs (Sumathipala et al., 2019). Yu et al. used Bi-Random Walks on the lncRNA functional similarity network and disease network to predict LDAs (Yu et al., 2017). Yu et al. (2020) constructed a data fusion model called Attributed Heterogeneous Network Fusion for LDA prediction (AHNF).

The second class of LDA prediction model is based on matrix factorization. Fu et al. proposed a LDA prediction model called MFLDA. MFLDA factored data from heterogeneous data sources into low-rank matrices based on matrix trivialization to discover and explore its intrinsic and shared structure (Fu et al., 2018). Wu et al. constructed a GAMCLDA model by encoding local graph structures and features. The graph convolution network was used to encode the features of this map structure and nodes to learn the potential factorial vectors of lncRNAs and diseases. In addition, the inner product of lncRNA factor vectors and disease factor vectors was used as a decoder to reconstruct the LDA matrix (Wu et al., 2020). Gao et al. (2021) constructed a multi-label fusion collaborative matrix decomposition approach to predict LDAs. Wang et al. (2020) developed a weighted matrix factorization model on multi-relational data to predict LDAs. Liu et al. (2021) introduced a weighted graph regularized collaborative matrix factorization (WGRCMF) method to predict LDAs.

The third class of LDA prediction model is based on machine algorithms. Machine learning methods focus on gaining insights into features and imbalanced labels. Chen et al. formulated Laplace regularized least squares method to predict LDAs (called LRLSLDA) in a semi-supervised learning framework, which is the first machine learning-based methods to predict LDAs (Chen et al., 2015). However, for LRLSLDA, parameter optimization is a challenge. Later, Chen et al. combined lncRNA functional similarity with the LRLSLDA-LNCSIM prediction model and enhanced its performance by introducing similarity scores for predicting gene-disease associations (Huang et al., 2016). In addition, Lan et al. implemented a LDAP model based on SVM bagging by combining disease similarity and lncRNA similarity (Lan et al., 2017). Yao et al. constructed a computational model called RFLDA to identify associations based on feature selection by integrating the experiment-supported associations among lncRNA, miRNA, disease, disease semantic similarity and lncRNA functional similarity (Yao et al., 2020). Xuan et al. have developed a collection of convolutional neural networks-based lncRNA-disease prediction models, including CNNLDA (Xuan et al., 2019a), LDAPred (Xuan et al., 2019b), GCNLDA (Xuan et al., 2019c) and CNNDLP (Xuan et al., 2019d). The CNNLDA developed an analysis of the associations between lncRNA and disease using convolutional neural networks that combined semantic and functional similarity as well as lncRNA-disease associations, miRNA-disease associations and lncRNA-miRNA interactions (Xuan et al., 2019a). The LDAPred integrated a convolutional neural network and information flow propagation, combining associations, interactions, similarity structures and topological structures between lncRNAs, miRNAs and diseases (Xuan et al., 2019b). The GCNLDA is based on the graph convolutional network and convolutional neural network to obtain locally integrated topological information within the lncRNA-disease-microRNA networks (Xuan et al., 2019c). By combining disease similarity, lncRNA similarity, miRNA-disease association and lncRNA-miRNA interactions, CNNDLP learned the attention and the low-dimensional network representation of the lncRNA-disease pairs (Xuan et al., 2019d). Wei et al. developed a method (LDICDL) that denoised lncRNA and disease features with an autoencoder, and used the matrix decomposition algorithm to test for potential disease-lncRNA association (Lan et al., 2022). Fan et al. proposed an lncRNA-disease prediction method that implemented convolutional matrices with conditional random fields and attention mechanisms for learning the embeddings of nodes for scoring latent associations between lncRNAs and diseases (Fan et al., 2022). Wu et al. proposed a method that combined extra trees with multi-layer graph embedding aggregation to predict LDAs (Wu Q. W. et al., 2021). Cui et al. proposed a novel model based on bipartite local model with nearest profile-based association inferring to predict LDAs (Cui et al., 2020).

These methods described above have achieved good prediction performance, but they also have some limitations. The biological network-based approach was affected by the scarcity of known LDA data; For the matrix factorization-based approach, the combination of model parameters is a very complex and necessary procedure; For the machine learning-based approach, feature processing and the impact of imbalanced data is a challenge. In this paper, we proposed a novel LDA prediction model based on geometric complement heterogeneous information and random forest (GCHIRFLDA in short). Firstly, the geometric complementation of LDA matrix was implemented by integrating the information of lncRNA-miRNA and miRNA-disease association information. Secondly, a low-dimensional feature space was extracted from the obtained LDA matrix by using an autoencoder, which combined Jaccard similarity coefficient and Gaussian interaction profile kernel similarity. Finally, a random forest classifier was trained on the constructed sample set to score potential lncRNA-disease associations. The AUC and AURP under five-fold cross-validation demonstrated that the GCHIRFLDA had a better performance than several state-of-the-art LDA prediction models, and the case studies on stomach cancer and colon cancer indicated that the GCHIRFLDA had excellent ability in identifying disease-associated lncRNAs.

## 2 Materials and methods

### 2.1 Representation of lncRNA-disease associations , miRNA-disease associations and lncRNA-miRNA interactions

LncRNA-disease associations (LDA), miRNA-disease associations (MDA) and lncRNA-miRNA interactions (LMI) were obtained from previous reports (Fu et al., 2018). The following l , d and m denote the number of lncRNA, disease and miRNA, respectively. The LDAs are represented by a 240 × × 412 adjacency matrix $LD_{i\times j}\in LD^{l\times d}$ , l is rows represent lncRNAs and d is columns represent diseases. For each element $LD_{i,j}$ , its value is equal to one if lncRNA i is related to disease j ; otherwise, its value is equal to 0. Similarly, the MDAs are represented by a 495 × × 412 adjacency matrix $MD_{i\times j}\in MD^{m\times d}$ , m is rows represent miRNAs and d is columns represent diseases. For each element $MD_{i,j}$ , its value is equal to one if miRNA i is related to disease j ; otherwise, its value is equal to 0. The LMIs are represented by a 240 × × 495 adjacency matrix $LM_{i\times j}\in LM^{l\times m}$ , l is rows represent lncRNAs and m is columns represent diseases. For each element $LM_{i,j}$ , its value is equal to one if lncRNA i is related to miRNA j ; otherwise, its value is equal to 0.

### 2.2 Calculation of jaccard similarity of disease and lncRNA

Calculation of similarity of disease and lncRNA is an important step in LDAs predicting process. So far, there are many ways to calculate similarity, such as disease semantic similarity, disease cosine similarity, lncRNA functional similarity and lncRNA cosine similarity. In this work, we combine the Jaccard similarity coefficient which is complementary to the binary matrix and the Gaussian interaction profile kernel similarity which encodes the non-linear vectors in the LDA matrix. By experimental research on different similarity measures, we found that the fusion of these two kinds of similarity can greatly improve the performance of the LDA prediction model. Therefore, we chose Jaccard similarity and Gaussian interaction profile kernel similarity for LDA prediction in this work. Thank you again for your comment. The Jaccard similarity coefficient (Jaccard, 1908) of disease was calculated by LDA matrix by Eq. 1:

$$\begin{array}{c} JDS\left(i,j\right)=\frac{LD\left(:,i\right)\cap^{\text{​}}LD\left(:,j\right)}{LD\left(:,i\right)\cup^{\text{​}}LD\left(:,j\right)} \end{array}$$ (1)

In Eq. 1, LD(:,i) is the *i*-th column vector of the LDA matrix, which represents the association feature of disease i ; $LD\left(:,i\right)\cap^{\text{​}}LD\left(:,j\right)\;$ represents the number of lncRNAs that are associated with both disease i and disease j ; $LD\left(:,i\right)\cup^{\text{​}}LD\left(:,j\right)$ represents the sum of the number of lncRNAs associated with the disease i and disease j .

Similarly to disease, the Jaccard similarity of lncRNA can be calculated by LDA matrix by Eq. 2:

$$\begin{array}{c} JFS\left(i,j\right)=\frac{LD\left(i,:\right)\cap^{\text{​}}LD\left(j,:\right)}{LD\left(i,:\right)\cup^{\text{​}}LD\left(j,:\right)} \end{array}$$ (2)

In Eq. 2, LD(i,:) is the i-th row vector of the LDA matrix, which represents the association feature of lncRNA *i*; $LD\left(i,:\right)\cap^{\text{​}}LD\left(j,:\right)$ represents the number of diseases that are associated with both lncRNA *i* and lncRNA *j*; $LD\left(i,:\right)\cup^{\text{​}}LD\left(j,:\right)$ represents the sum of the number of diseases associated with the lncRNA *i* and lncRNA *j*.

### 2.3 Calculation of Gaussian interaction profile kernel similarity of disease and lncRNA

The Gaussian interaction profile kernel similarity (Chen, 2015b) $GIP_{lnc}\left(l_{i},l_{j}\right)$ between lncRNA $l_{i}$ and lncRNA $l_{j}$ was calculated by Eq. 3:

$$\begin{array}{c} \{\begin{array}{c} GIP_{lnc}\left(l_{i},l_{j}\right)=\exp\left(-\lambda {\left\|LD\left(i,:\right)-LD\left(j,:\right)\right\|}^{2}\right)\\ \lambda =\tilde{\lambda }/\left(\frac{1}{l}\sum_{i=1}^{l}{\left\|l_{i}\right\|}^{2}\right) \end{array}\;\;\;\;\;\;\;\;\; \end{array}$$ (3)

From the above equation, the Gaussian interaction profile kernel similarity matrix of lncRNA can be obtained. LD(i,:) and LD(j,:) represents i -th and *j*-th row of LDA matrix respectively, $\tilde{\lambda }$ controls the kernel bandwidth, in this work, we set $\tilde{\lambda }$ to 1.

Similarly, the Gaussian interaction profile kernel similarity matrix of disease $GIP_{dis}\left(d_{i},d_{j}\right)$ can be obtained by Eq. 4.

$$\begin{array}{c} \{\begin{array}{c} GIP_{dis}\left(d_{i},d_{j}\right)=\exp\left(-\lambda {\left\|LD\left(:,i\right)-LD\left(:,j\right)\right\|}^{2}\right)\\ \lambda =\tilde{\lambda }/\left(\frac{1}{d}\sum_{i=1}^{d}{\left\|d_{i}\right\|}^{2}\right) \end{array}\;\;\;\; \end{array}$$ (4)

In Eq. 4, LD(:,i) and LD(:,j) represents *i*-th and *j*-th column of LDA matrix respectively, $\tilde{\lambda }$ controls the kernel bandwidth, in this work, we set $\tilde{\lambda }$ to 1.

### 2.4 Fusing different similarities for lncRNA and disease

In this paper, we used the maximum value method to merge lncRNA Gaussian interaction profile kernel similarity and lncRNA Jaccard similarity into LFJ similarity and fuse disease Gaussian interaction profile kernel similarity and disease Jaccard similarity into DSJ similarity by Eqs. 5, 6, respectively.

$$\begin{array}{c} LFJ\;similarity=\{\begin{array}{c} GIP_{lnc}\left(l_{i},l_{j}\right)\;\;if\;GIP_{lnc}\left(l_{i},l_{j}\right)\geq JFS\left(i,j\right)\\ JFS\left(i,j\right)\;\;\;\;\;\;\;\;\;\;\;\;\;\;\;\;\;\;\;\;\;\;\;\;\;\;\;\;\;\;\;\;otherwise \end{array} \end{array}$$ (5)

$$\begin{array}{c} DSJ\;similarity=\{\begin{array}{c} GIP_{dis}\left(d_{i},d_{j}\right)\;if\;GIP_{dis}\left(d_{i},d_{j}\right)\;\geq JDS\left(i,j\right)\\ JDS\left(i,j\right)\;\;\;\;\;\;\;\;\;\;\;\;\;\;\;\;\;\;\;\;\;\;\;\;\;\;\;\;\;\;\;\;\;\;\;otherwise \end{array}\; \end{array}$$ (6)

### 2.5 Geometric complement for lncRNA-disease associations matrix

**FIGURE 1:**
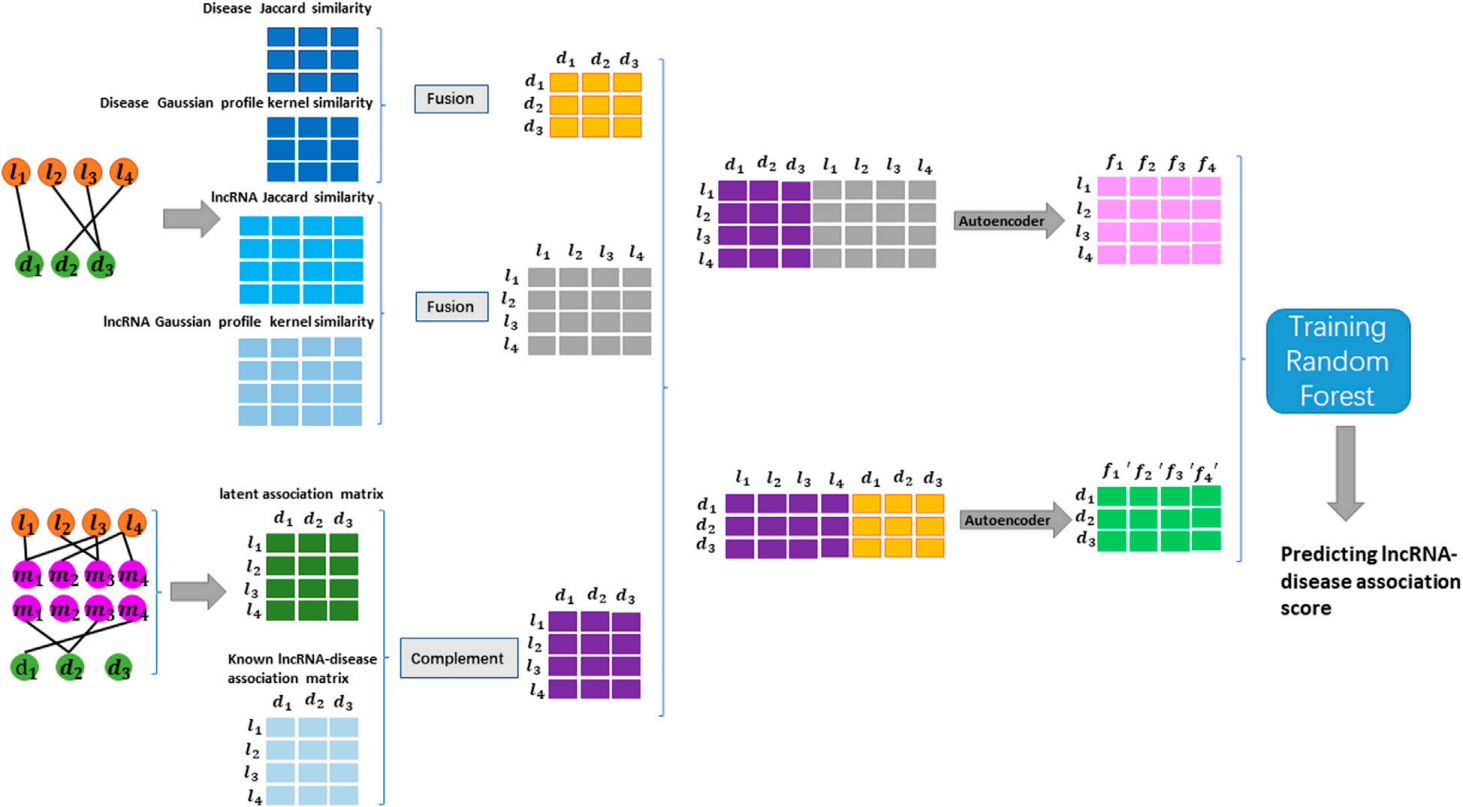
The flowchart of constructing the GCHIRFLDA model.

The process of constructing the GCHIRFLDA model is divided into three steps (see Figure 1): 1) geometric complement for LDA matrix; 2) feature representation and extraction; 3) random forest classifier training and LDA prediction. Next, we will introduce the process of constructing the GCHIRFLDA model in detail.

Inspired by Francesco et al.‘s and Yin et al.‘s method (Wang et al., 2021; Yin et al., 2022), from the previous data source, we multiplied the LMI matrix with the MDA matrix and then divided the [*i, j*]-th element of the result by the *i*-th row of the LMI matrix and the *j*-th column of the MDA matrix to represent the potential LDA matrix by Eq. 7:

$$\begin{array}{c} LMD\left(\text{i},\text{j}\right)=\frac{LM\left(i,:\right)\cdot MD\left(:,j\right)}{{\left\|LM\left(i,:\right)\right\|}_{1}+{\left\|MD\left(:,j\right)\right\|}_{1}} \end{array}$$ (7)

The fusion matrix of LDA was obtained by taking the maximum value of the potential LDAs computed above and the original LDA matrix in the *i*-th row and *j*-th column by Eq. 8.

$$\begin{array}{c} LD_{\text{new}}\left(i,j\right)=\max\left(LD\left(i,j\right),\text{LMD}\left(\text{i},\text{j}\right)\right) \end{array}$$ (8)

In this way, the original LDA matrix can be geometrically complemented.

### 2.6 Feature representation and extraction

For the obtained geometric complement matrix, each row represents the feature vector of lncRNA and each column represents the feature vector of disease. We combine the *i*-th row of the geometric complement matrix and the *i*-th row of the similarity fusion matrix of lncRNA to form a new feature vector of the *i*-th lncRNA. Similarly, we combine the *j*-th column of the geometric complement matrix and the *j*-th column of the similarity fusion matrix of disease to form a new feature vector of the *j*-th disease. Finally, each lncRNA and disease is represented as a 652-dimensional feature vector.

Autoencoder is an unsupervised neural network model and has a good performance in data denoising and dimensionality reduction. In the GCHIRFLDA model, we employee autoencoder to compress feature space of lncRNA and disease. We set hidden layer to learn the high-dimensional feature space of the input data so that the hidden layer can reconstruct the original input data (Schmidhuber, 2015; Ji et al., 2021).

In this work, we use an autoencoder with an input layer, a dense layer, an output layer and a fully-connected layer with an activation function sigmoid. The learning process of the noise-reducing encoder is to minimize the error between the reconstructed data and the original data. As a result, each lncRNA, which is originally represented by a 652-dimensional feature vector, is finally compressed into 256-dimensional by autoencoder. Similarly, each disease, which is originally represented by a 652-dimensional feature vector, is finally compressed into 256-dimensional by autoencoder. MSE (mean squared error) is used as model loss evaluation by Eq. 9:

$$\begin{array}{c} \mathrm{loss}=\frac{1}{n}\sum {\left(Y_{input}-Y_{output}\right)}^{2} \end{array}$$ (9)

In Eq. 9, $Y_{input}$ is the original input data, and $Y_{output}$ is the decoded and reconstructed data.

### 2.7 Random forest classifier training and lncRNA-disease associations prediction

To train the GCHIRFLDA model, the experiment-supported 2697 LDAs in the original LDA matrix were used as positive samples; the remaining lncRNA-disease pairs that were not validated by biological experiments were used as unlabeled samples. To maintain the balance of the training set, an equal number of unlabeled samples were randomly selected from the unlabeled samples as negative samples. The negative samples and the positive samples were combined into the training sample set which consisted of 5394 samples.

For accurately predicting potential LDAs, we employed random forest (RF) for LDA prediction in the GCHIRFLDA model. Random forest is an ensemble machine learning model which combines bagging and random features to add extra diversity of the decision tree model and finally uses a voting method to combine the prediction results of multiple base classifiers (Breiman, 2001). RF has many advantages: 1) it can process a variety of data types, including qualitative data or quantitative data; 2) it has high classification accuracy; 3) it has good robustness for noise data and data with missing values; 4) it has ability to analyze complex interactions between features. In recent years, RF has been widely used in a variety of classification and prediction problems, including differential expression analysis of microarray data, miRNA-disease association prediction, etc. In this work, we have carried out experimental research on six different classifiers, including SVM and Xgboost. Considering AUC, AUPR, Recall and other indicators, the performance of RF classifier is the best. Therefore, RF was chosen as the final classifier in our prediction model. RF has two important parameters, namely the number of randomly selected features (*mtry*) and the number of trees (*ntree*). These parameters have a great impact on the performance of random forest classification model. Here, we set *mtry* and *ntree* by the default value. Then, by the obtained prediction model, all unconfirmed lncRNA-disease pairs are scored, and the closer the score is to 1, the more likely it is that lncRNA is associated with the disease.

## 3 Results

### 3.1 Feature dimension analysis of lncRNA and disease

For LDA prediction, the dimensionality of the training sample set has an obvious impact on the accuracy of the prediction model. On the one hand, for a smaller number of features of lncRNAs and diseases, more features are not learned, which leads to under-fitting of the model. On the other hand, for a larger number of features, more time is spent and the model performance will not yet be greatly improved or even over-fitting will occur. Therefore, we used the experimental method to determine the appropriate feature dimension. Specifically, we use autoencoder to compress the dimensions of feature space into 16, 32, 64, 128, 256, and 512 respectively, and the feature dimension that makes the prediction performance of the model the highest is adopted. Table 1 shows the AUC obtained under five-fold cross-validation by different dimensional features, from which one can see that the maximum of AUC is reached when the feature dimension of both lncRNAs and diseases is 256, so we set the feature dimension of extracted lncRNAs and diseases by autoencoder to be 256.

**TABLE 1:** The AUCs under different lncRNA/disease feature dimension.

Dimension	**16**	**32**	**64**	**128**	**256**	**512**
**16**	0.9576	0.9724	0.9768	0.9782	0.9750	0.9724
**32**	0.9492	0.9753	0.9775	0.9809	0.9804	0.9788
**64**	0.9577	0.9760	0.9791	0.9833	0.9842	0.9826
**128**	0.9561	0.9764	0.9808	0.9872	0.9884	0.9877
**256**	0.9539	0.9736	0.9804	0.9874	**0.9897**	0.9889
**512**	0.9109	0.9711	0.9793	0.9880	0.9891	0.9890

### 3.2 Performance comparison between random forest and other classifiers

In order to obtain better performance of the GCHIRFLDA model, we compared RF classifier with several classical classifiers, including extreme gradient boosting (Xgboost) (Chen and Guestrin, 2016), C50 (Kuhn, 2013), Gradient Boosting Decision Tree (GBDT) (Ye et al., 2009), SVM (Lan et al., 2017) and LightGBM (Zhang et al., 2021). In this work, we used the average AUC, AUPR, Recall, F1-score and Accuracy based on five-fold cross-validation as evaluation criterion for the six classifiers.

**FIGURE 2:**
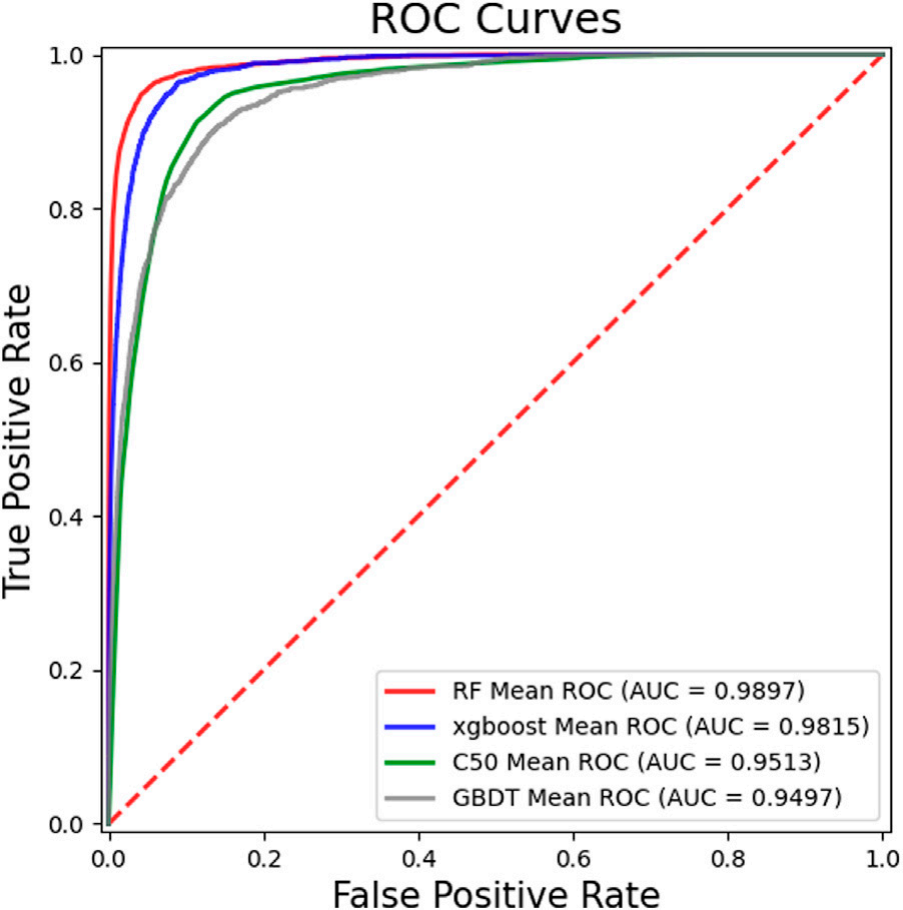
The ROC Curves of different classifiers in the GCHIRFLDA model.

**FIGURE 3:**
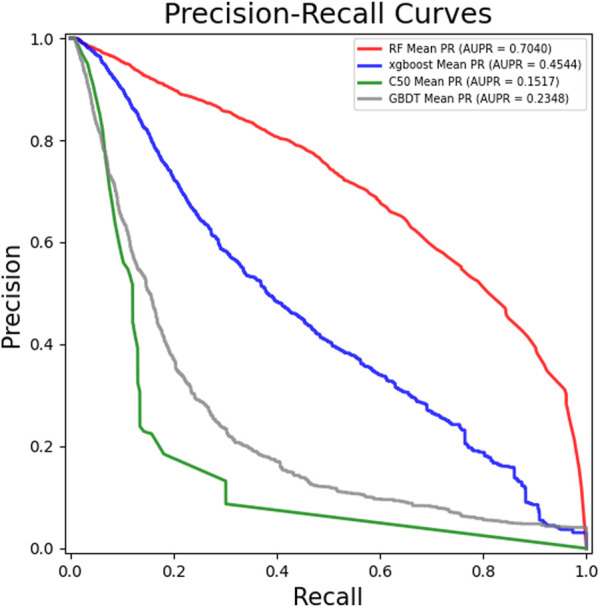
The Precision-Recall Curves of different classifiers in the GCHIRFLDA model.

Figure 2 showed the ROC curves and AUCs of different classifiers, from which one can see that the AUC values of RF, Xgboost, C50 and GBDT are 0.9897, 0.9814, 0.98959 and 0.9497, respectively. Figure 3 showed the PR curves and AUPRs of four classifiers, the AUPR values of RF, Xgboost, C50 and GBDT are 0.704, 0.4505, 0.1607 and 0.2336, respectively. Table 2 showed the AUC, AUPR, Recall, F1-score and Accuracy of six classifiers. As one can see from Table 2, all five metrics of RF is the largest among the six classifiers. The results of the experiments suggested that RF outperformed the other five classifiers for LDA prediction. There, RF was finally determined as the final classifier in the GCHIRFLDA model.

**TABLE 2:** The performance comparison of different classifiers in the GCHIRFLDA model.

Classifier	AUC	AUPR	Recall	Accuracy	F1-score
Xgboost	0.9815	0.4544	0.9523	0.9182	0.9523
RF	**0.9897**	**0.7040**	**0.9673**	**0.9317**	**0.9597**
C50	0.9513	0.1517	0.9340	0.8724	0.9265
GBDT	0.9497	0.2348	0.8942	0.8701	0.9253
SVM	0.9832	0.5826	0.9243	0.9313	0.9595
LightGBM	0.9832	0.5250	0.9428	0.9215	0.9541

### 3.3 Performance comparison between GCHIRFLDA and other lncRNA-disease associations prediction models

To evaluate the prediction performance of the GCHIRFLDA model, we compared it with seven state-of-the-art LDA prediction models, including GAERF (Wu Q.-W. et al., 2021), CNNLDA (Xuan et al., 2019a), GCNLDA (Xuan et al., 2019c), MFLDA (Fu et al., 2018), Ping’s method (Ping et al., 2019) and SIMLDA (Lu et al., 2018). The AUCs and AUPRs of all LDA prediction models are listed in Table 3. Figure 3 showed the ROC curves for these LDA prediction models.

**TABLE 3:** The AUCs and AUPRs of different LDA prediction models.

Method	AUC	AUPR
GCHIRFLDA	**0.990**	**0.704**
GAERF	0.980	0.491
GCNLDA	0.959	0.223
CNNLDA	0.952	0.251
LDAP	0.863	0.166
MFLDA	0.626	0.066
Ping’s Method	0.871	0.219
SIMCLDA	0.746	0.095

**FIGURE 4:**
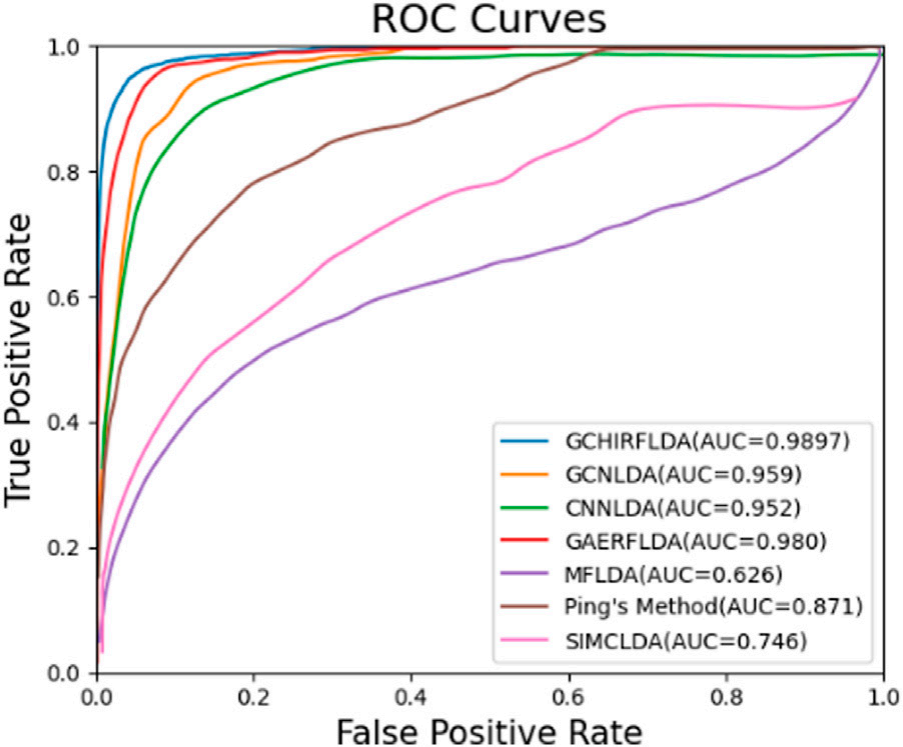
The ROC Curves of different LDA prediction models.

From Table 3 and Figure 4, one can see that the AUC and AUPR of the GCHIRFLDA model are maximal among all LDA prediction models, which achieved 0.990 and 0.704, respectively. In term of AUC, our model achieved 0.990 which was 0.99%, 3.23%, 3.96%, 58.19%, 13.63%, and 32.67% higher than GAERF, GCNLDA, CNNLDA, MFLDA, Ping’s method and SIMCLDA, respectively. In term of AUPR, our model achieved 0.704 which was 43.38%, 215.79%, 180.47%, 966.67%, 221.46%, 634.38% higher than GAERF, GCNLDA, CNNLDA, MFLDA, Ping’s Method and SIMCLDA, respectively. According to the results of cross validation experiments, our GCHIRFLDA model has better LDA prediction ability.

### 3.4 Case studies

To further validate the prediction ability of the GCHIRFLDA model, we conducted case studies on two most common cancers, colon cancer and stomach cancer. We used the GCHIRFLDA to score all the unlabeled lncRNA-disease pairs, and selected the top 20 lncRNAs most likely to be associated with stomach cancer and colon cancer respectively according to the score. Finally, the predicted stomach cancer-associated and colon cancer-associated lncRNAs by the GCHIRFLDA model were validated by data from Lnc2Cancer v3.0 (Ning et al., 2016), LncRNADisease v2.0 (Bao et al., 2019) and some published research literature.

Colon cancer is the third most common cancer worldwide and the fourth leading cause of cancer-related death. The incidence of colon cancer has increased dramatically in China because of a shift in our habits as a society (Xue et al., 2015). In this work, we used the GCHIRFLDA to predict colon cance-associated lncRNAs. As a result, the top 20 predicted lncRNAs associated with colon cancer and the provenances of the evidence are shown in Table 4. As one can see from Table 4, 17 predicted lncRNAs have been confirmed by records included in the Lnc2Cancer (v3.0) or LncRNADisease (v2.0) or published literature. For example, Wan et al. showed that the overexpressing of CDKN2B-AS1 exhibited accelerated proliferation in colon cancer (Wan et al., 2013). Xu et al. reported the tumor suppressor B-cell linker (BLNK) was reduced in expression *via* MIR17HG, which resulted in an increase in invasion and migration of colorectal cancer cells (Xu et al., 2019).

**TABLE 4:** The top 20 colon cancer-related lncRNA candidates predicted by the GCHIRFLDA model.

lncRNA	Rank	Evidence
CDKN2B-AS1	1	Lnc2Cancer 3.0& LncRNADisease v2.0
PVT1	2	Lnc2Cancer 3.0& LncRNADisease v2.0
UCA1	3	Lnc2Cancer 3.0& LncRNADisease v2.0
NEAT1	4	Lnc2Cancer 3.0& LncRNADisease v2.0
KCNQ1OT1	5	Lnc2Cancer 3.0
XIST	6	Lnc2Cancer 3.0& LncRNADisease v2.0
GAS5	7	Lnc2Cancer 3.0& LncRNADisease v2.0
SPRY4-IT1	8	Lnc2Cancer 3.0& LncRNADisease v2.0
MIR17HG	9	Literature (Xu et al., 2019)
TUG1	10	Lnc2Cancer 3.0& LncRNADisease v2.0
BANCR	11	Lnc2Cancer 3.0& LncRNADisease v2.0
HOTTIP	12	Lnc2Cancer 3.0& LncRNADisease v2.0
BCYRN1	13	LncRNADiseasev2.0
HNF1A-AS1	14	Lnc2Cancer 3.0
AFAP1-AS1	15	Lnc2Cancer 3.0
HULC	16	Lnc2Cancer 3.0
TUSC7	17	Lnc2Cancer 3.0
KIRREL3-AS3	18	unknown
LSINCT5	19	unknown
NPTN-IT1	20	unknown

In the digestive tract, stomach cancer is one of the most prevalent malignancies (Gu et al., 2017). The identification of new biomolecular markers of stomach cancer is essential for treatment and diagnosis. In this work, we used the GCHIRFLDA to predict stomach cancer-associated lncRNAs. As a result, the top 20 predicted lncRNAs associated with colon cancer and the provenances of the evidence are shown in Table 5. As seen in Table 5, 18 predicted lncRNAs have been confirmed by records included in the Lnc2Cancer (v3.0) or LncRNADisease (v2.0) or published literature. For example, Feng et al. revealed that KCNQ1OT1 inhibited stomach cancer cell progression *via* regulating miR-9 and LMX1A expression (Feng et al., 2020); Wu et al. found the high expression of lncRNA-CCAT2 indicated poor prognosis of stomach cancer and promoted cell proliferation and invasion (Wu et al., 2017). Consequently, the case studies on colon cancer and stomach cancer showed that GCHIRFLDA was an excellent predictor.

**TABLE 5:** The top 20stomach cancer-related lncRNA candidates predicted by the GCHIRFLDA model.

lncRNA	Rank	Evidence
MALAT1	1	Lnc2Cancer 3.0& LncRNADisease v2.0
XIST	2	Lnc2Cancer 3.0& LncRNADisease v2.0
NEAT1	3	Lnc2Cancer 3.0& LncRNADisease v2.0
CCAT2	4	Lnc2Cancer 3.0& LncRNADisease v2.0
TUG1	5	Lnc2Cancer 3.0& LncRNADisease v2.0
KCNQ1OT1	6	Lnc2Cancer 3.0
HOTTIP	7	Lnc2Cancer 3.0& LncRNADisease v2.0
WT1-AS	8	Lnc2Cancer 3.0& LncRNADisease v2.0
HNF1A-AS1	9	Lnc2Cancer 3.0& LncRNADisease v2.0
HULC	10	Lnc2Cancer 3.0& LncRNADisease v2.0
MIR17HG	11	Literature (Bahari et al., 2015)
CRNDE	12	Lnc2Cancer 3.0& LncRNADisease v2.0
NPTN-IT1	13	Lnc2Cancer 3.0& LncRNADisease v2.0
LINC00675	14	Lnc2Cancer 3.0
KIRREL3-AS3	15	unknown
TP53COR1	16	unknown
BCYRN1	17	Lnc2Cancer 3.0
HOTAIRM1	18	Lnc2Cancer 3.0
AFAP1-AS1	19	LncRNADisease v.2.0
LINC01133	20	Lnc2Cancer 3.0

## 4 Conclusion

In this work, we proposed a geometric complement heterogeneous information and random forest-based approach for predicting LDAs (named GCHIRFLDA). Firstly, the potential LDA matrix is constructed by integrating the LMIs and MDAs with the original LDA matrix. Then, the Jaccard similarity and the Gaussian interaction profile similarity of lncRNA and disease are combined to represent features of lncRNA and disease. Next, a low-dimensional feature space is extracted by using autoencoder. Finally, RF is employed as the classifier to predict potential LDAs. In conclusion, the AUC and AUPR comparison with other LDA prediction models based on five-fold cross-validation and the case studies show that our model has better LDA prediction performance.

Although the GCHIRFLDA model has a good performance, it still has some limitations. Firstly, the lack of data verified by biological experimental is a big shortcoming for computational models. Secondly, randomly selecting the unknown lncRNA-disease pairs as negative samples may incorrectly classify potential positive samples as negative samples, which may affect the prediction performance. Finally, only the heterogeneous information of miRNAs is introduced in this work, and in the future, more biological information will be fused to improve the performance of the LDA prediction model.

## Data Availability

The original contributions presented in the study are included in the article/supplementary material, further inquiries can be directed to the corresponding author.
